# Alternative Pathways of Acetylcholine Release in the Colon: Role of High‐Affinity Choline Transporters

**DOI:** 10.1111/nmo.70280

**Published:** 2026-03-07

**Authors:** A. Martinez‐Daunis, B. Yordanova, S. Traserra, P. Vergara, M. Jimenez

**Affiliations:** ^1^ Department of Cell Biology, Physiology and Immunology and Neurosciences Institute Universitat Autònoma de Barcelona Barcelona Spain; ^2^ Centro de Investigación Biomédica en Red de Enfermedades Hepáticas y Digestivas (CIBEREHD) Barcelona Spain

**Keywords:** acetylcholine, choline transporter, colon, high affinity, organic cation transporter, smooth muscle contraction

## Abstract

**Background:**

Cholinergic neuromuscular transmission is central to gastrointestinal (GI) motility and is traditionally attributed to calcium‐dependent, vesicular acetylcholine (ACh) release from enteric neurons. However, non‐quantal, calcium‐independent mechanisms—possibly involving transporter‐mediated ACh efflux—may also contribute to cholinergic signaling.

**Aim:**

To investigate both classical and alternative mechanisms of ACh release in the colon, focusing on the potential role of non‐vesicular, transporter‐dependent pathways in modulating smooth muscle contractility.

**Methods:**

Experiments were performed on full‐thickness and epithelium‐depleted rat colonic muscle strips. Neostigmine, a reversible acetylcholinesterase inhibitor, was used to enhance cholinergic mechanisms. A panel of pharmacological agents—including tetrodotoxin (TTX selective blocker of Na^+^ channels), ω‐conotoxin GVIA (Ca^2+^ *N*‐type channel blocker), Hemicholinium (choline transporter inhibitor), corticosterone (OCTs inhibitor), and hexamethonium (nicotinic receptor antagonist)—was applied to differentiate neural, non‐neural, and transporter‐mediated contributions to ACh release.

**Key Results:**

Neostigmine‐induced contractions were preserved in epithelium‐depleted strips, following neural blockade with TTX and ω‐conotoxin GVIA. Hemicholinium concentration‐dependently attenuated these contractions, suggesting involvement of high‐affinity choline transporters operating in reverse mode. In contrast, corticosterone and hexamethonium had negligible effects, arguing against substantial roles for OCTs and nicotinic transmission.

**Conclusions and Inferences:**

These findings support the existence of a non‐vesicular, transporter‐dependent cholinergic signaling mechanism in the colon. This alternative pathway may contribute to the regulation of colonic motility and represents a novel target in GI motility modulation.

## Key Points

1. Neostigmine‐induced contractions in rat colon persist despite neural blockade, indicating a non‐vesicular mechanism of acetylcholine release.
2. Hemicholinium significantly inhibits these contractions, implicating high‐affinity choline transporters operating in reverse as a key mediator of this alternative cholinergic pathway.
3. High‐affinity choline transporters could provide a novel pathway for acetylcholine release originating from neuronal sources.

## Graphical abstract

Mechanisms of Acetylcholine release, including typical and atypical release under neuronal blockade. Left panel illustrates the classical (quantal) cholinergic transmission and the right panel the mechanism described in the present study. High‐Affinity Choline Transporter (HCT), Acetylcholine (ACh), Acetylcholinesterase (ACE), and Tetrodotoxin (TTX).

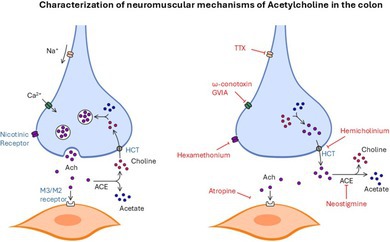

## 1 Introduction

Cholinergic neuromuscular transmission plays a crucial role in regulating smooth muscle contractions within the gastrointestinal (GI) tract. Acetylcholine (ACh), the primary neurotransmitter involved in this process, is released from cholinergic neurons and acts on muscarinic receptors (primarily M2 and M3) located on smooth muscle cells and other post‐junctional cells such as Interstitial cells of Cajal (ICC) and PDGFRα^+^ cells that are electrically coupled [1, 2]. The release of ACh from nerve terminals is most commonly a calcium‐dependent process. Calcium influx through voltage‐gated calcium channels triggers the fusion of synaptic vesicles with the presynaptic membrane, leading to the subsequent release of ACh into the neuromuscular junction. This process is essential for activating cholinergic receptors on post‐junctional cells, initiating contraction, and coordinating GI motility. At the neuromuscular junction and possibly in the autonomic nervous system, this type of release is often referred to as quantal release, where neurotransmitters are released in discrete, quantized amounts, each associated with the fusion and neurotransmitter release of a single vesicle [3].

Emerging research has suggested the existence of calcium‐independent release mechanisms for Ach, which can occur through pathways that do not require calcium influx [4, 5, 6]. In these mechanisms, neurotransmitters are released in a more diffuse, non‐quantized manner, and this type of release is known as non‐quantal release. Non‐quantal release may be mediated by the reverse‐mode operation of transporters such as organic cation transporters (OCTs) and choline transporters (CHTs). These transporters may facilitate the release of Ach without the need for synaptic vesicle fusion or calcium influx, potentially contributing to more sustained or localized cholinergic signaling. This could influence smooth muscle contractility and modulate GI motility in a non‐traditional manner. Corticosterone is an OCT3 blocker and nuclear glucocorticoid stimulator, and less efficient blocking OCT1 and OCT2. Hemicholinium is a CHTs inhibitor.

Neostigmine, a reversible inhibitor of AChE, has been widely used in experimental settings to prolong the action of Ach. By inhibiting AChE, which typically breaks down Ach at the neuromuscular junction, neostigmine increases the availability of Ach and thereby potentiates smooth muscle contractions. Neostigmine is used in clinics to recover from paralytic ileus [7] and it is also used in refractory colonic psudoobstruction [8]. The pharmacological effect is probably due to an enhancement of the neural cholinergic system since it is well recognized that neostigmine enhances neural‐mediated contractions. However, two recent studies performed ex vivo, have shown that neostigmine can increase gastric and colonic contractility despite neural blockade with tetrodotoxin (TTX), suggesting that calcium‐independent, non‐quantal release of Ach can occur in cholinergic neurons, potentially providing an alternative mechanism for modulating smooth muscle activity [2, 9].

In addition to neural release, non‐neural release of Ach has also been recognized in many systems [10, 11]. Many cell types express choline acetyl transferase (ChAT) or carnitine acetyl transferase (CarAT) the enzymes responsible for Ach synthesis and therefore they are potential sources of non‐neural Ach. The release of Ach by non‐neural cells has been classified as the non‐neural cholinergic system (NNCS). The epithelium is able to release Ach possibly through OCT [12]. Recent data suggest that potential release of Ach comes from Tuft cells that are located within the epithelial layer of the GI tract [13]. Tuft cells are specialized chemosensory cells that produce and release Ach independently of the classical cholinergic neurons. This non‐neural release may contribute to the broader cholinergic signaling in the GI tract and could have implications for local motility regulation and smooth muscle function [13, 14].

This study aims to explore both calcium‐dependent and calcium‐independent release mechanisms of Ach, with a particular focus on the possibility that OCTs and CHTs, acting in reverse mode, may participate in the release of Ach at the neuromuscular junction. By employing various experimental protocols, this study seeks to investigate the contributions of these transporters to cholinergic signaling and their potential role in modulating GI motility.

## 2 Materials and Methods

### 2.1 Ethics Statement

The experimental procedures were approved by the Ethics Committee of the Universitat Autònoma de Barcelona (approval code MJF‐eut/01) and adhered to the European Community Council Directive 2010/63/EU of the European Parliament on the protection of animals used for scientific purposes.

### 2.2 Animals and Tissue Preparation

Thirty‐seven Sprague–Dawley rats (20 females and 17 males), aged 7 to 10 weeks, were housed under controlled conditions: temperature (22°C ± 2°C), humidity (55% ± 10%), a 12‐h light/dark cycle, and ad libitum access to food and water. The rats were euthanized by decapitation without prior anesthesia. This procedure was approved by the institutional animal ethics committee and was used to avoid anesthetic‐induced interference with intestinal motility and cholinergic signaling. The colon was rapidly excised and immediately immersed in carbogenated (95% O_2_ and 5% CO_2_) Krebs solution. The mesenteric fat was removed, and the colon was longitudinally opened along the mesenteric border. The tissue was then pinned onto a Sylgard‐coated base with the mucosal side facing upward. The mid‐colon section used was defined according to previously published anatomical criteria [15]. In some experiments, full‐thickness tissue was used for mechanical studies, while in others, the mucosal and submucosal layers were removed. In some cases, the contractile response was measured in either the circular or longitudinal orientation (see experiments in Table 1).

**TABLE 1:** Experimental protocols.

Experiment	Protocol: Sequential drug addition	Colonic tissue
1	Non‐nitregic, non‐purinergic conditions EFS responses TTX (10^−6^ M), ω‐CTX (10^−7^ M). Atropine (10^−9^ to 10^−6^ M)	Circular muscle: Isolated^a^
2	TTX (10^−6^ M) Neostigmine (10^−5^ M) Atropine (10^−6^ M)	Circular muscle: Full thickness Isolated^a^
3	TTX (10^−6^ M) Neostigmine (10^−5^ M) Hemicholinium (10^−5^ to 10^−3^ M) or Corticosterone (10^−7^ to 10^−6^ M) or Hexamethonium (10^−6^ to 10^−5^ M). Atropine (10^−6^ M)	Isolated circular muscle^a^ Isolated longitudinal muscle^b^
4	ω‐CTX (10^−7^ M). Neostigmine (10^−5^ M) Hemicholinium (10^−5^ to 10^−3^ M) Atropine (10^−6^ M)	Isolated circular muscle^a^ Isolated longitudinal muscle^b^
5	TTX (10^−6^M) Hemicholinium (10^−5^ to 10^−3^ M) or Corticosterone (10^−5^ M) or Hexamethonium (10^−5^ M). Neostigmine (10^−5^ M) Atropine (10^−6^ M)	Isolated circular muscle^a^

^a^ Isolated circular muscle. Tissue in circular orientation devoid of mucosa and submucosa.

^b^ Isolated longitudinal muscle. Tissue in longitudinal orientation, devoid of mucosa and submucosa.

### 2.3 Mechanical Studies

Muscle strips (4 × 10 mm) were studied in a 10‐mL organ bath containing Krebs solution, maintained at 37°C ± 1°C and continuously carbogenated (95% O_2_ and 5% CO_2_) to ensure optimal oxygenation. A tension of 1 g was applied, and the tissues were equilibrated for 1 h to allow stabilization of spontaneous contractile activity. Mechanical responses were recorded using an isometric force transducer (UF‐1, Harvard Apparatus) connected to a computer via an amplifier. Data acquisition was performed at a sampling rate of 25 Hz using Data 2001 software (Panlab), interfaced with an A/D converter.

### 2.4 Experiments

Each experiment is summarized in Table 1.

#### 2.4.1 Experiment 1

Electrical field stimulation (EFS) through two platinum electrodes was applied at regular intervals (every 100 s). Stimulation parameters were set at 30 V, 50 Hz, with a 0.4 ms pulse duration and a train duration of 300 ms. Initially, responses were assessed under control conditions. Tissues were then incubated with L‐NNA (1 mM) and MRS2500 (1 μM) to block the inhibitory pathway and isolate the excitatory pathway (non‐nitrergic, non‐purinergic conditions). The amplitude of the contractile responses was evaluated before and after drug incubation. In this protocol, TTX, ω‐Conotoxin GVIA (ω‐CTX), and atropine (ranging from 10^−9^ to 10^−6^ M) were used.

#### 2.4.2 Experiment 2

To block neural‐mediated excitatory contractions, tissues were incubated for 15 min with TTX (10^−6^ M). In the presence of TTX, tissues were then incubated with neostigmine (10^−5^ M). These experiments were conducted on both full‐thickness strips and strips devoid of mucosa and submucosa. Atropine (10^−6^ M) was added at the end of the experiment to block neostigmine responses.

#### 2.4.3 Experiment 3

In these experiments, hemicholinium (10^−5^ to 10^−3^ M), corticosterone (10^−7^ to 10^−6^ M), or hexamethonium (10^−6^ M to 10^−5^ M) were added after neostigmine. Atropine (10^−6^ M) was used at the end of the experiment. These experiments were performed on strips devoid of mucosa and submucosa in both circular and longitudinal orientations.

#### 2.4.4 Experiment 4

In these experiments, the effect of hemicholinium was tested in the presence of ω‐CTX (10^−7^ M). Atropine (10^−6^ M) was used at the end of the experiment. Strips were studied in both circular and longitudinal orientations.

#### 2.4.5 Experiment 5

Since a certain run‐down of the responses was observed after neostigmine in all experiments, the experiments were repeated by first incubating with TTX 10^−6^ and hexamethonium, corticosterone, or hemicholinium, followed by the addition of neostigmine (10^−5^ M) after the incubation of these drugs. Atropine (10^−6^ M) was used at the end of the experiment.

### 2.5 Measurements

When EFS was applied (Experiment 1), the amplitude of the response was measured before and after drug addition. Data was normalized to 100% (before drug addition), and the percentage reduction after drug addition was calculated.

For the remaining experiments (Experiments 2 to 5), contractile activity was quantified by calculating during 2–3 min and the area under the curve (AUC) relative to the baseline. The AUC (g min^−1^) was considered 100% before drug addition. After each drug addition, the AUC was recalculated, and the increase or decrease in the contractile response relative to basal activity was measured.

### 2.6 Statistics

Paired *t*‐test was used to compare the drug effect before and after drug addition. Two‐way ANOVA or one‐way ANOVA test was used to compare the drug effect in each experimental protocol.

### 2.7 Drugs and Solutions

The composition of the Krebs solution was (in mmol/L): glucose 10.10, NaCl 115.48, NaHCO_3_ 21.90, KCl 4.61, NaH_2_PO_4_ 1.14, CaCl_2_ 2.50, and MgSO_4_ 1.16 bubbled with a mixture of 5% CO_2_–95% O_2_ (pH 7.4).

The following drugs were used: TTX (CAS number: 1078; Tocris), Atropine (CAS number: 51‐55‐8; Merck), hemicholinium‐3 (CAS number: 312‐45‐8: Merck), Corticosterone (CAS number: 50‐22‐6 Merck), hexamethonium chloride (CAS number: 200‐465‐1: Merck), Neostigmine bromide (CAS number: 114‐80‐7; Merck). Nω‐Nitro‐L‐arginine (L‐NNA; CAS number: 2149‐70‐4; Merck), MRS 2500 tetraammonium salt (CAS number: 630103‐23‐0, Tocris), ω‐CTX (CAS number: 106375‐28‐4; Tocris).

All stock solutions were prepared by dissolving the drugs in distilled water, except for corticosterone, which was dissolved in ethanol 96%. L‐NNA required sonication to be dissolved in Krebs solution.

### 2.8 Correlation With Single‐Cell RNA Analysis

Our pharmacological data were correlated with data obtained with single‐cell RNA analysis available at (the single cell portal: https://singlecell.broadinstitute.org/single_cell [16]). In this study the mRNA of different cell types was identified at single cell resolution.

## 3 Results

### 3.1 Experiment 1. Neural Release of Acetylcholine

**FIGURE 1:**
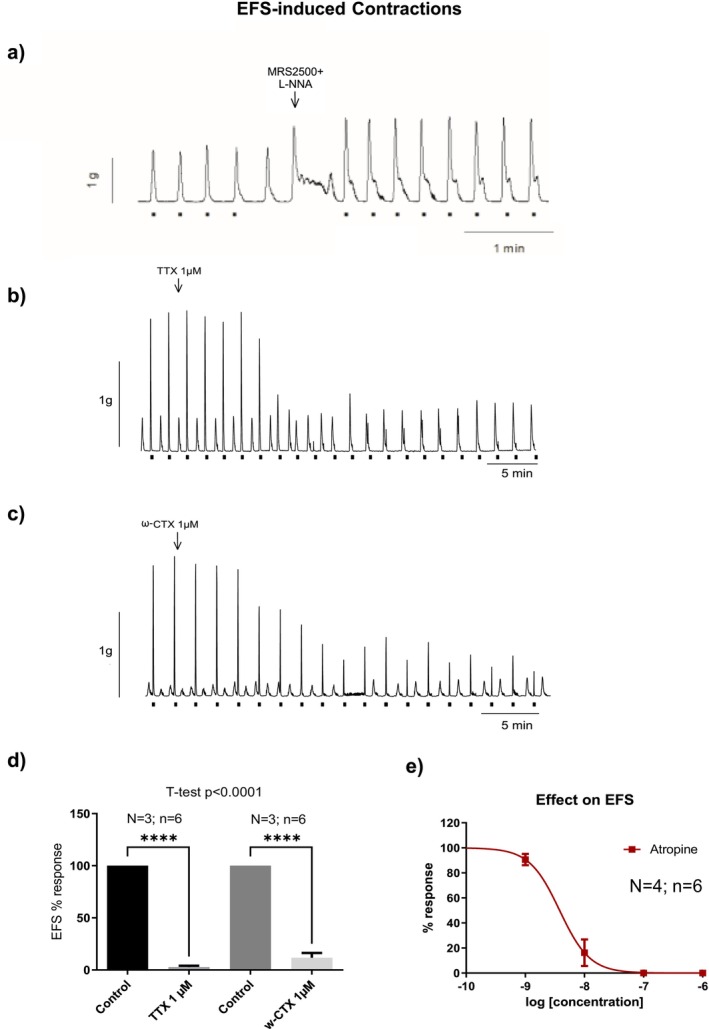
Effect of TTX, ω‐conotoxin and atropine on EFS‐induced contractions (dots in the tracing) in the colonic smooth muscle. In the representative tracing shown in panel (a), the marked increase in basal tone corresponds to the addition of L‐NNA (1 mM) and MRS2500 (1 μM), used to block inhibitory nitrergic and purinergic neural inputs and thereby isolate excitatory neural responses. Tissue was incubated with L‐NNA 1 mM and MRS2500 1 μM to isolate neural mediated excitatory responses. Neural responses were blocked by TTX 10^−6^ M, ω‐CTX 10^−6^ M (b, c, d) and by Atropine 10^−8^ M (e). Data were normalized (i.e., 100%) to the electrical field stimulation amplitude before drug addition. Data are expressed as mean ± SEM, *n* = 6 tissue strips from 3 to 4 rats per group. Paired *t*‐test: ****p < 0.0001 compared to control response.

To isolate the cholinergic component of neuromuscular transmission, experiments were conducted under non‐nitrergic, non‐purinergic conditions using L‐NNA (1 mM) and MRS2500 (1 μM). Under these pharmacological conditions, EFS elicited a sharp contraction, which was reduced in a concentration‐dependent manner by atropine and completely abolished by TTX (1 μM) and ω‐conotoxin GVIA (0.1 μM) (Figure 1). These results indicate that EFS‐induced contractions were both neural‐ and calcium‐dependent, mediated by muscarinic receptor activation in post‐junctional cells (Figure 1).

### 3.2 Experiment 2. Full‐Thickness vs. Isolated Circular Muscle Responses

**FIGURE 2:**
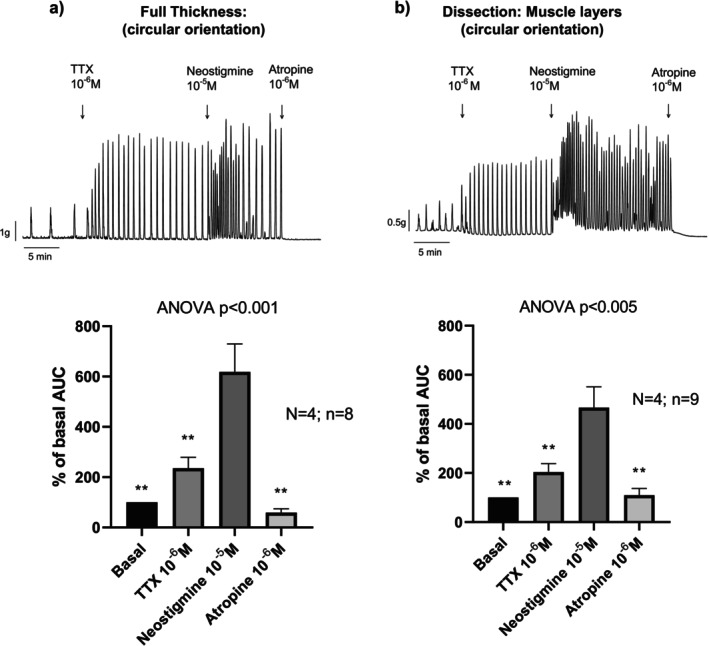
Effect of neostigmine on colonic contractility in the presence of neural blockade (TTX). (a) Full thickness muscle (b) Muscle layers without mucosa and submucosa. Data were normalized (i.e., 100%) to the Area Under the Curve before TTX addition. Data are expressed as mean ± SEM, *n* = 8–9 tissue strips from 4 rats per group. ANOVA paired test followed by Dunnett's post hoc test: **p < 0.01 compared to neostigmine response.

In a second set of experiments, the ACE blocker, neostigmine, was used to enhance spontaneous contractility. TTX was applied to block neural mediated cholinergic responses. Given the possibility that cholinergic activity could originate from the epithelium, experiments were conducted in preparations with and without the mucosa and submucosa layers (Figure 2). In both conditions, neostigmine increased contractile activity, which was markedly reduced by atropine. These findings suggest activation of a cholinergic mechanism that is, at least in part, independent of epithelial contribution (Figure 2).

### 3.3 Experiment 3. Neostigmine Responses: Effects of Hemicholinium‐3, Corticosterone, and Hexamethonium

**FIGURE 3:**
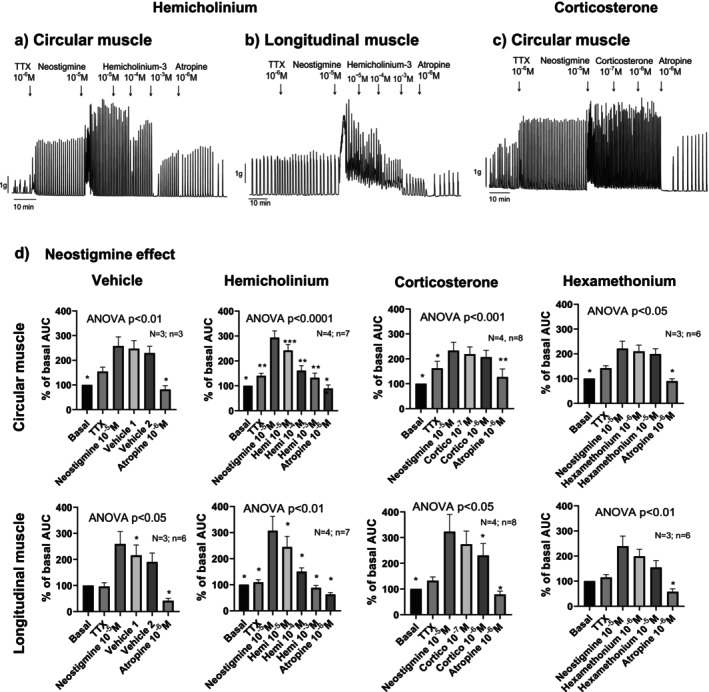
Effect of Hemicholinium‐3, Corticosterone, and Hexamethonium on neostigmine‐induced responses in circular and longitudinal colon contractility. Representative tracings (a, b and c) and histograms (d) showing the effect of the sequential addition of each drug in each muscle layer. ANOVA paired test followed by Dunnett's post hoc test: *p < 0.05; **p < 0.01, ***p < 0.001 compared to neostigmine response. Data are expressed as mean ± SEM, *n* = 3–8 tissue strips from 3 to 4 rats per group.

In a third set of experiments, the same protocol was applied to both circular and longitudinal muscle strips devoid of epithelium (Figure 3). In both muscle orientations, neostigmine increased spontaneous contractile activity even in the presence of TTX, indicating that the effect is independent of neural input and not confined to a specific muscle layer.

Following neostigmine application, incubation with hemicholinium‐3 led to a concentration‐dependent reduction in the contractile response. This suggests that the neostigmine‐induced release of Ach probably involves the CHTs. In contrast, hexamethonium—an antagonist of nicotinic receptors—had no effect on the response, consistent with a non‐nicotinic mechanism.

Corticosterone (1 μM), used to inhibit OCTs, slightly reduced neostigmine responses in longitudinal muscle but had no significant effect in circular muscle. A similar, modest reduction in longitudinal muscle contraction was observed with vehicle alone, likely reflecting a natural rundown of the neostigmine response over time (Figure 3). This observation prompted the design of Experiment 5 to address and control for this time‐dependent decline.

### 3.4 Experiment 4. Effect of Hemicholinium‐3 in the Presence of ω‐CTX

**FIGURE 4:**
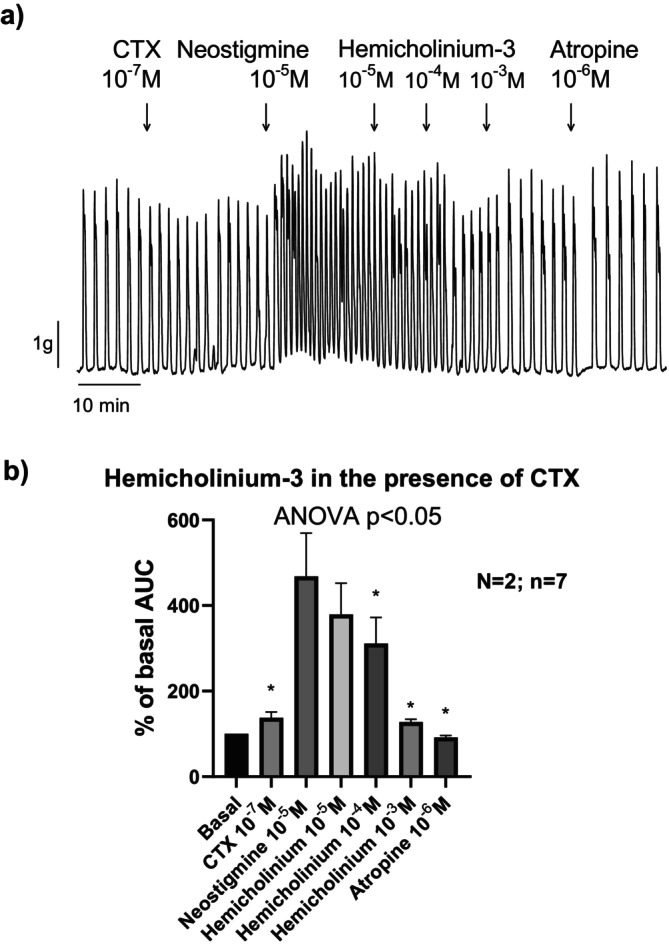
Effect of Hemicholinium‐3 on neostigmine‐induced contractions in the presence of the N‐type calcium channel blocker ω‐CTX. Representative tracing (a) and histogram (b) showing the contractility after a sequential drug addition. ANOVA paired test followed by Dunnett's post hoc test: *p < 0.05 compared to neostigmine response. Data are expressed as mean ± SEM, *n* = 7 tissue strips from 2 rats.

Given that neural Ach release is calcium‐dependent, we evaluated the effects of neostigmine in the presence of ω‐CTX, which selectively blocks N‐type calcium channels and thereby inhibits neurogenic cholinergic transmission, as shown in Experiment 1. In the presence of ω‐CTX, neostigmine continued to enhance contractile activity in both circular and longitudinal muscle layers (Figure 4). These findings suggest that the neostigmine‐induced responses are independent of calcium influx through N‐type calcium channels. Subsequent addition of hemicholinium led to a concentration‐dependent reduction of the neostigmine‐induced responses (Figure 4).

### 3.5 Experiment 5. Effect of Neostigmine Following Pre‐Incubation With Hemicholinium‐3, Corticosterone, and Hexamethonium

As noted in Experiment 4, neostigmine responses tend to diminish over time. To control for this rundown effect, tissues were pre‐incubated with potential inhibitors before the addition of neostigmine.

**FIGURE 5:**
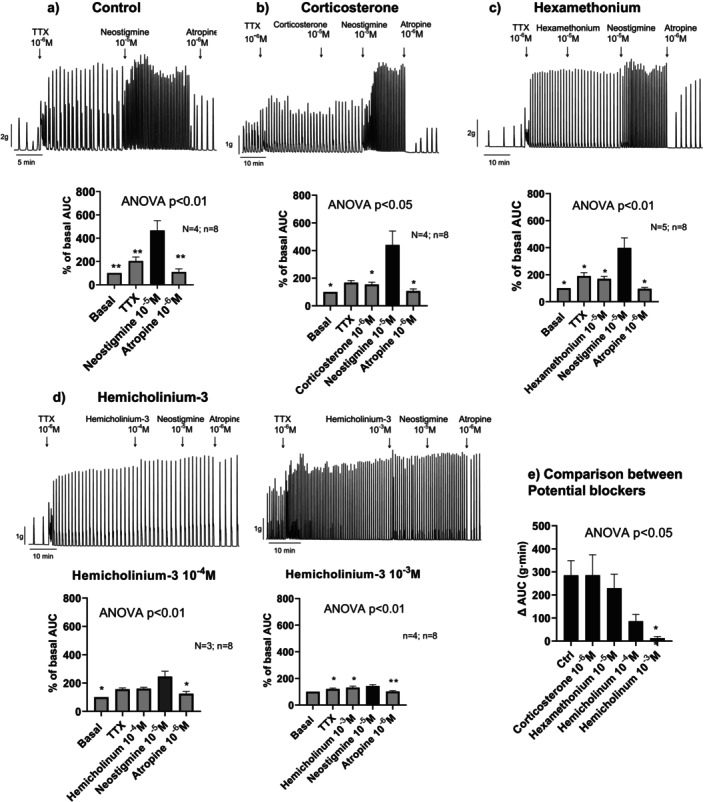
Representative tracings (top) and histograms (bottom) illustrating the effect of Neostigmine on colonic contractility after incubation with different drugs. (a) Control incubated with TTX; (b) Incubation with TTX and corticosterone; (c) Incubation with TTX and hexamethonium; (d) Incubation with TTX and Hemicholinium‐3 (10^−4^ M and 10^−3^ M); (e) Comparison of the different potential blockers. Notice that for this comparison the increase in AUC (∆ AUC) after each drug addition was calculated in each experimental protocol. Data are expressed as mean ± SEM, *n* = 8 tissue strips from 3 to 5 rats per group. Statistical analysis was performed using a repeated ANOVA (a–d) or non repeated ANOVA (e) followed by Dunnett's post hoc test: *p < 0.05; **p < 0.01 compared to the neostigmine response under control conditions.

Pre‐incubation with corticosterone (1 μM), or with hexamethonium (10 μM), did not significantly alter the contractile response to neostigmine. These findings indicate that neither OCTs nor nicotinic receptors play a major role in mediating the observed cholinergic activity under these conditions. However, pre‐incubation with hemicholinium‐3 led to a concentration‐dependent inhibition of neostigmine‐induced contractions (Figure 5).

### 3.6 Correlation With Single Cell RNA Analysis

In experiments previously published where mouse single cell RNA analysis was performed [16]. ChAT was mainly expressed in Neurons and in a subset of Tuft cells that also express Avil and DLCK1 (Figure S1). Interestingly, the gene responsible for the CHTs (SLC5 A7) is strongly expressed in enteric neurons, suggesting that the effect of hemicholinium‐3 is associated with a block of the CHTs. Figure S1 serves as comparative support rather than direct experimental evidence.

## 4 Discussion

Cholinergic neuromuscular transmission, which traditionally relies on calcium‐mediated vesicular ACh release, is essential for GI motility.

However, in other systems, experimental evidences suggest that non‐quantal, calcium‐independent mechanisms may also contribute to ACh signaling, potentially via transporter‐mediated pathways [4, 5, 17, 18]. Moreover, non‐neural cholinergic systems are mainly located in epithelial Tuft Cells. Other potential non‐neural cholinergic systems might also exist. For example, cells such as ICC express ChAT in the intramuscular layer of the bladder [19], but this is not the case in the GI tract since they do not express ChAT in the GI tract and therefore they are not potential intramuscular sources of non‐neural ACh. Preliminary experiments from our lab performed in the ChAT‐Cre/Ai9 transgenic mouse mainly show two potential sources of ChAT. This line expresses the Cre‐recombinase enzyme under the control of the ChAT gene promoter and therefore endogenous neurons of both plexuses and Tuft cells expressing DLCK1 are endogenously marked [20]. These data correlate with single cell RNAm analysis [15] where ChAT was exclusively located in enteric neurons and Tuft cells. Suggesting that a non‐neural source of Ach might exist or alternatively atypical neural release of Ach is present in the enteric nervous system as it has been described in other systems [18, 21, 22]. Accordingly, in this study we assessed both classical and alternative ACh release mechanisms in rat colonic tissue using neostigmine, a reversible acetylcholinesterase inhibitor, to enhance endogenous cholinergic contractions. Experiments were conducted on full‐thickness and epithelium‐depleted colonic muscle strips to distinguish between epithelial and non‐epithelial sources of ACh. In both tissues, neostigmine induced an increase in the contractions suggesting that the mechanism is probably not associated with cholinergic tuft cells or other potential sources of ACh at the epithelial level. Alternatively, the contraction induced by neostigmine might be due to neural cells. It is important to note that experiments were conducted employing pharmacological blockers such as TTX or ω‐CTX that effectively blocked neural cholinergic contractions. In experiments where radiolabeled ACh has been measured, TTX does not decrease basal ACh release, suggesting that the mechanism is independent of neural action potentials [23]. The results found in the present work occur in both muscle layers as it has recently been shown in human colonic tissue [2]. When interpreting the effects of TTX across the different experimental protocols, it should be noted that non‐nitrergic and non‐purinergic conditions were selectively established only in Experiment 1. In subsequent experiments, inhibitory neural pathways—including nitrergic, purinergic and potentially VIPergic inputs—were not pharmacologically blocked. Therefore, the increase in contractile activity observed after TTX in some preparations (Figures 2, 3, 4, 5) may partially reflect removal of an ongoing inhibitory neural tone, resulting in disinhibition of smooth muscle activity and/or unmasking of intrinsic myogenic contractions. This phenomenon has been previously described in GI smooth muscle and should be taken into account when interpreting TTX‐resistant responses. This study provides compelling evidence supporting the existence of a calcium‐independent, non‐quantal mechanism of ACh (ACh) release in the rat colon. While classical cholinergic signaling is dependent on calcium influx through voltage‐gated channels and synaptic vesicle fusion, our findings reveal that neostigmine‐induced enhancement of contractility persists even under conditions that block neural conduction and vesicular release. Specifically, the resistance of these contractile responses to TTX and ω‐CTX indicates a mechanism independent of action potential propagation and N‐type calcium channel activity.

The observed effects of hemicholinium‐3, a CHTs inhibitor, provide critical insight into the non‐vesicular pathways involved. Previous experiments have shown that hemicholinium‐3 decreased both choline uptake and consequently ACh release in the ENS. However, neuromuscular responses were not affected [23]. It is important to note that long incubations are needed to observe these effects. The consistent and concentration‐dependent attenuation of neostigmine‐induced contractions by hemicholinium‐3 suggests that reverse‐mode operation of CHT may facilitate ACh efflux under certain conditions. Similar results have been documented in other parts of the autonomic nervous system [4, 5, 18]. This notion aligns with earlier hypotheses that CHTs, typically involved in choline uptake, may under pharmacological or physiological stress act in reverse to mediate ACh release. It is important to notice that the CHTs are exclusively expressed in neurons (see mRNA expression in Figure S1) and therefore its pharmacological effect should be attributed to neural Ach.

Conversely, the lack of significant effect of corticosterone and hexamethonium argues against a major role for OCTs or nicotinic receptors in this setting. While corticosterone has been proposed as an OCT inhibitor [22], its minimal impact here, even with pre‐incubation, indicates that OCTs are not primary contributors to the non‐quantal release of ACh in the rat colon under these experimental conditions. Similarly, the ineffectiveness of hexamethonium suggests that the contractile responses are not mediated by nicotinic receptor activation. This result is pharmacologically relevant since a potential interaction between Hemicholinium‐ and nicotinic receptors has been previously reported [24].

Interestingly, the neostigmine‐induced responses were similar in both full‐thickness and mucosa‐depleted preparations, suggesting that epithelial sources of ACh, such as tuft cells, do not significantly contribute to the observed cholinergic activity. However, it is possible that ACh released by tuft cells might also contribute to motility changes through the ENS. Further studies are needed to define the contribution of cholinergic tuft cells to ENS modulation.

While the present study provides functional evidence, some limitations should be acknowledged. The conclusions are primarily based on pharmacological modulation of contractile responses, and ACh release was not directly quantified. Although this approach is widely used to infer cholinergic mechanisms and the pharmacological profile observed is consistent with non‐vesicular ACh release mediated by the CHTs, the absence of direct biochemical measurements prevents definitive confirmation of ACh efflux and its precise cellular origin. Future studies combining functional recordings with direct assessment of ACh levels will be useful to further substantiate the proposed mechanism. A second limitation is the potential off‐target effects of Hemicholinium‐3, such as nicotinic receptor blockade. We assessed the effect of hexamethonium and observed no effect; however, other off‐target effects cannot be excluded. Additionally, the comparison with single‐cell RNA analysis was performed using mouse colon, whereas the present study used rat colon. The extent to which differences in receptor and transporter distribution between species may be relevant remains unknown. It would therefore be interesting to perform experiments on human tissue to investigate whether this mechanism is present in the human colon, particularly since in our previous experiments, contractions induced by neostigmine occurred even in the presence of neural blockade [2].

## 5 Conclusion

The implications of these findings are substantial. Non‐vesicular ACh release could represent a physiologically relevant mechanism for maintaining baseline smooth muscle tone or modulating local motility. It may also play a compensatory role in pathological conditions where neural transmission is impaired. Clinically, this pathway could be targeted to modulate GI motility disorders, such as paralytic ileus or colonic pseudo‐obstruction, where neostigmine has already shown efficacy.

## Author Contributions

A. Martinez‐Daunis and B. Yordanova contributed equally to this work. B. Yordanova, S. Traserra and A. Martinez‐Daunis, performed the experimental procedures and carried out the data analysis. A. Martinez‐Daunis drafted the initial manuscript. M. Jimenez conceived the study, designed the experimental protocols, and contributed to manuscript writing. P. Vergara supervised the project, secured funding, and provided scientific guidance. All authors critically revised the manuscript and approved the final version.

## Conflicts of Interest

The authors declare no conflicts of interest.

## Supporting information


**Figure S1:** Dot‐plot showing the % and mean expression of different genes (Right column) in different cell types (Top). The dot plot shows the % of cells from a particular group that express on gene and the scaled mean expression is the mean intensity of expression. Data published in single cell portal obtained from mouse large intestine (https://singlecell.broadinstitute.org/single_cell/study/SCP1038/the‐human‐and‐mouse‐enteric‐nervous‐system‐at‐single‐cell‐resolution?label=PEMN_1&genes=Avil%2CDclk1%2CChat%2CSlc5a7%2CSlc22a1%2CSlc22a2%2CSlc22a13%2CChrm3&cluster=mli.tsne2.txt&spatialGroups=‐‐&annotation=LABEL‐‐group‐‐cluster&subsample=100000) [25]. Avil and Dclk1 are the mRNA of the gene encoding Advilin and doublecortin like Kinase 1 which both are markers of Tuftt cells in rodents. ChAT is the mRNA of genes encoding Choline acetyltransferase. Notice its expression in Neurons and Tuft Cells. Slc5a7 is the RNAm of the gene encoding the High affinity choline transporter 1 which is highly expressed in neurons. Slc22a1, 2 and 13 are the mRNA of the genes encoding OCTs 1, 2 and 3 respectively. Chrm3 is the mRNA of the gene encoding cholinergic receptor muscarinic 3.

## Data Availability

The data that support the findings of this study are available on request from the corresponding author. The data are not publicly available due to privacy or ethical restrictions.
