# Supplementary figures and images for: Alternative Pathways of Acetylcholine Release in the Colon: Role of High‐Affinity Choline Transporters
Source: Neurogastroenterol Motil. 2026 Mar 7;38(3):e70280. doi: 10.1111/nmo.70280 (PMC12967756; doi:10.1111/nmo.70280)

## Slide 1
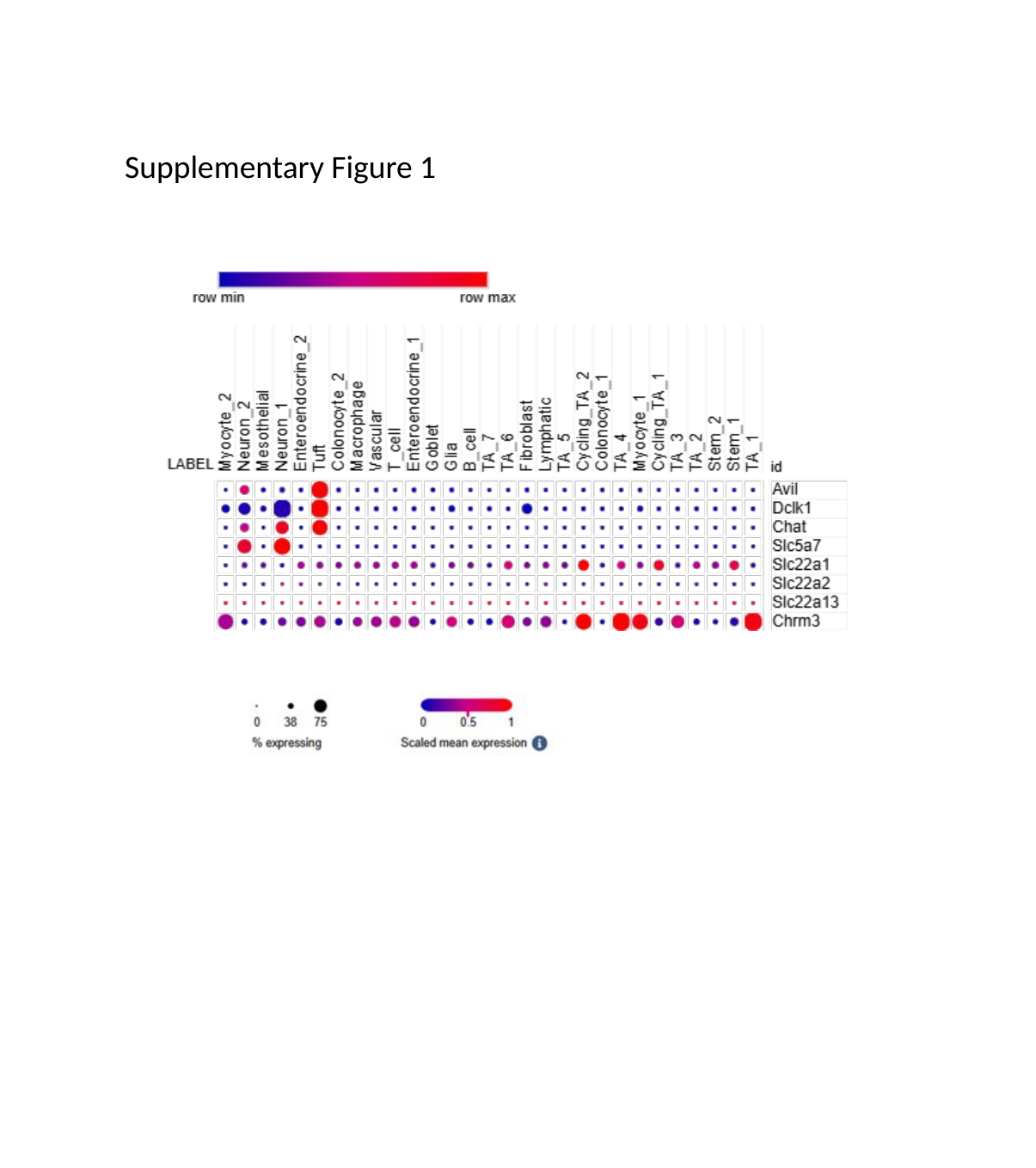

Supplementary Figure 1

Supplement: Supplementary file 1 — Figure S1: Dot‐plot showing the % and mean expression of different genes (Right column) in different cell types (Top). The dot plot shows the % of cells from a particular group that express on gene and the scaled mean expression is the mean intensity of expression. Data published in single cell portal obtained from mouse large intestine (https://singlecell.broadinstitute.org/single_cell/study/SCP1038/the‐human‐and‐mouse‐enteric‐nervous‐system‐at‐single‐cell‐resolution?label=PEMN_1&genes=Avil%2CDclk1%2CChat%2CSlc5a7%2CSlc22a1%2CSlc22a2%2CSlc22a13%2CChrm3&cluster=mli.tsne2.txt&spatialGroups=‐‐&annotation=LABEL‐‐group‐‐cluster&subsample=100000) [25]. Avil and Dclk1 are the mRNA of the gene encoding Advilin and doublecortin like Kinase 1 which both are markers of Tuftt cells in rodents. ChAT is the mRNA of genes encoding Choline acetyltransferase. Notice its expression in Neurons and Tuft Cells. Slc5a7 is the RNAm of the gene encoding the High affinity choline transporter 1 which is highly expressed in neurons. Slc22a1, 2 and 13 are the mRNA of the genes encoding OCTs 1, 2 and 3 respectively. Chrm3 is the mRNA of the gene encoding cholinergic receptor muscarinic 3. [file NMO-38-e70280-s001.pptx]
